# Rice lipid transfer protein, OsLTPL23, controls seed germination by regulating starch-sugar conversion and ABA homeostasis

**DOI:** 10.3389/fgene.2023.1111318

**Published:** 2023-01-16

**Authors:** Quanlin Li, Wenxue Zhai, Jiaping Wei, Yanfeng Jia

**Affiliations:** ^1^ Institute of Genetics and Developmental Biology, Chinese Academy of Sciences, Beijing, China; ^2^ State Key Laboratory of Aridland Crop Science, Gansu Agricultural University, Lanzhou, China

**Keywords:** seed germination, rice, OsLTPL23, soluble sugar, abscisic acid (ABA)

## Abstract

Seed germination is vital for ensuring the continuity of life in spermatophyte. High-quality seed germination usually represents good seedling establishment and plant production. Here, we identified OsLTPL23, a putative rice non-specific lipid transport protein, as an important regulator responsible for seed germination. Subcellular localization analysis confirmed that OsLTPL23 is present in the plasma membrane and nucleus. The knockout mutants of *OsLTPL23* were generated by CRISPR/Cas9-mediated genome editing, and *osltpl23* lines significantly germinated slower and lower than the Nipponbare (NIP). Starch and soluble sugar contents measurement showed that OsLTPL23 may have alpha-amylase inhibitor activity, and high soluble sugar content may be a causal agent for the delayed seed germination of *osltpl23* mutants. Transcript profiles in the germinating seeds exhibited that the abscisic acid (ABA)-responsive genes, *OsABI3* and *OsABI5*, and biosynthesis genes, *OsNCED1*, *OsNCED2*, *OsNCED3* and *OsNCED4*, are obviously upregulated in the *osltpl23* mutants compared to NIP plants, conversely, ABA metabolism genes *OsABA8ox1*, *OsABA8ox2* and *OsABA8ox3* are stepwise decreased. Further investigations found that *osltpl23* mutants displays weakened early seedling growth, with elevated gene expresssion of ABA catabolism genes and repressive transcription response of defence-related genes *OsWRKY45, OsEiN3, OsPR1a*, *OsPR1b* and *OsNPR1*. Integrated analysis indicated that *OsLTPL23* may exert an favorable effect on rice seed germination and early seedling growth *via* modulating endogenous ABA homeostasis. Collectively, our study provides important insights into the roles of *OsLTPL23*-mediated carbohydrate conversion and endogenous ABA pathway on seed germination and early seedling growth, which contributes to high-vigor seed production in rice breeding.

## Introduction

Seed vigor in plant determines seed germination, seedling emergence and growth, and seed storage ability under favorable or adverse environmental conditions (Sun et al., 2007; Finch-Savage and Bassel, 2016). Seed germination, to some extent, is synonymous with seed vigor (Finch-Savage and Bassel, 2016; Leprince et al., 2017), in which the germination rate is usually used as morphological indicator of seed viability (Wang et al., 2010). High-quality seed germination results in robust and healthy post-germination seedling growth, with the ability to withstand stressful environment (Foolad et al., 2007). Therefore, identification of the genes responsible for seed germination will contribute to develop high-vigor plant varieties and crop production.

The phytohormone ABA acts an indispensable roles in inhibiting seed germination and promoting seed dormancy (Penfield, 2017). The degradation of ABA content in seeds alleviates the ABA signaling pathway during seed imbibition (Preston et al., 2009). The reports from independent groups have demonstrated that the ABA-responsive transcription factors, *ABSCISIC ACID INSENSITIVE 3* (*ABI3*), *ABSCISIC ACID INSENSITIVE 4* (*ABI4*) and *ABSCISIC ACID INSENSITIVE 5* (*ABI5*), exert distinct roles in germinated seeds and early seedling growth. The loss-of-function alleles of *ABI3* reduces the ABA-mediated inhibition during seed germination and post-germination growth (Ding et al., 2014). Salinity-induced *ABI4* repress seed germination and post-germination growth by promoting ROS accumulation (Luo et al., 2021). *ABI5* enhances exogenous ABA-mediated developmental arrest from seed germination to vegetative growth (Lopez-Molina et al., 2001). Soluble sugar is another important regulator in seed germination and post-germination seedling development in plants (Dekkers et al., 2004; Zhu et al., 2009; Zhao et al., 2018). The germination delay of plant seeds by exogenous glucose results from the suppressive ABA catabolism (Dekkers et al., 2004; Zhu et al., 2009), whereas exogenous ABA application inhibits seed germination through locally restricting glucose availability of the embryonic hypocotyl (Xue et al., 2021; Deng et al., 2020). In early seedling growth, the inhibitory effect of exogenous glucose is accomplished by through *ABI4*-mediated sugar-ABA signaling pathway (Arroyo et al., 2003; Bossi et al., 2009; Li et al., 2014a). However, the interaction of endogenous ABA and soluble sugar in plant seed germination and early seedling growth is still mysterious.

Plant non-specific lipid transfer proteins (nsLTPs) are small basic lipid-binding proteins, which precursors typically harbour an hydrophobic signal peptide in N-terminal and an internal hydrophobic molecules binding cavity forming by an conserved eight cysteine motif (8CM, C-Xn-C-Xn-CC-Xn-CXC-Xn-C-Xn-C) (Kader, 1996; Salminen et al., 2016; Meng et al., 2018; Fleury et al., 2019). In rice, the nsLTPs, reportedly, reside in the plasma membrane (Li et al., 2020; Zhao et al., 2020; Chen et al., 2022), cytoplasm (Fujino et al., 2008; Li et al., 2020) and nucleolus (Fujino et al., 2008), with the function in abiotic stress (Guo et al., 2013; Zhao et al., 2020), pollen development (Zhang et al., 2010; Li et al., 2020; Tao et al., 2020; Chen et al., 2022), plant height (Li et al., 2014b; Ding et al., 2014), as well as embryo development and seed germination (Wang et al., 2015). There are only two investigations of nsLTPs on early seedling growth and seed germination in rice. The *OsLTPL159* allele distributed in different rice groups confers a distinct seedling cold tolerance (Zhao et al., 2020), and *OsLTPL36* contributes to rice embryo development, seed quality, seed germination and early seedling growth (Wang et al., 2015). Whereas, the association between ABA, soluble sugar, and nsLTPs-mediated seed germination and post-germination seedling growth awaits disclosure.

In this work, we characterized a rice plasma membrane- and nucleus-localized nsLTP, OsLTPL23. Site-specific mutation of *OsLTPL23* resulted in an impeded seed germination and weakened seedling growth. Starch-sugar analysis implied that OsLTPL23 protein serves as an alpha-amylase inhibitor in seed. Prompted by the messenger RNA levels of *OsABIs, OsNCEDs* and *OsABA8oxs*, endogenous ABA contributed to *OsLTPL23-*dependent phenotype in seed germination and early seedling growth. Summarily, these findings indicated that *OsLTPL23* promotes rice seed germination and post-germination seedling growth.

## Materials and methods

### Plant growth conditions

The germinated rice seeds were grown in a plant greenhouse at 30°C during 14 h daytime and 25°C with 10 h night under 70% humidity. All seeds were harvested at maturity stage, and stored at room temperature after drying at 42°C for 7 days.

### Vector construction and rice transformation

The konck-out vector Cas9-OsLTPL23-gRNA was generated as previously reported (Xu et al., 2014). The specific sgRNA sequences targeted for *OsLTPL23* were inserted into the pHUN4c12 vector digested with *Bsa* I-HF using T4 DNA ligase reaction (2011A; Takara, Japan). The resultant construct Cas9-OsLTPL23-gRNA was transformed into the *japonica* variety NIP through the *Agrobacterium* *tumefaciens* strain EH105-mediated stable transformation (Nishimura et al., 2006). Resistant rice calli were vigorously grown in hygromycin-containing medium, and finally transferred to regeneration medium to obtain transgenic plants.

### Transgenic-free mutants screening

To identify the *osltpl23* mutants, the genomic DNA was extracted from the leaves of hygromycin-resistant plants using CTAB method, and then used as template to perform PCR amplification with OsLTPL23-Cas9-detection primers (Supplementary Table S4). All the DNA sequences of PCR products from the above plants were directly determinate to identify Cas9-editing events using Sanger sequencing techniques. Homozygous mutants and heterozygotes were indicated as normal sequencing chromatograms carrying simple indels and superimposed sequencing chromatograms, respectively.

To determine transgenic-free mutants, the T1 generation plants of Cas9-cutting lines were analyzed by PCR amplification with HPT-specific primers (Supplementary Table S4). The Cas9-OsLTPL23-gRNA vector and genomic DNA of NIP were chosen as positive control and negative control, repectively. The *HPT*-negative mutation lines were marked as transgenic-free mutants.

### Seed germination

Fifty seeds per replicate of each *osltpl23* mutants and NIP were germinated on Petri dishes containing moistened paper towels at 26°C for 6 days. The seed germination criterion and germination rate determination were as stated in Wang et al. (2021). The seeds germination assay was conducted in triplicate.

### Quantitative reverse transcription-PCR (qRT-PCR) assay

Total RNA was isolated from the germinating seeds (12, 24, 48, and 72 h post imbibition (hpi)) and various tissues at two developmental stages (root, leaf and sheath of five-leaf stage seedlings; stem, flag leaf and spike of booting stage plants) of NIP, and two-week-old seedlings of NIP, *osltpl23-1* and *osltpl23-2* mutants using the TRIzol™ reagent (15596-026; Invitrogen, Carlsbad, California, United States). 1 μg total RNA was used to first-strand cDNA synthesis by the ReverTra Ace qPCR RT Master Mix with gDNA Remover (FSQ-301; TOYOBO, Osaka, Japan). The qRT-PCR was performed using TransStart^®^ Tip Green qPCR SuperMix (+Dye I) (AQ142-11; TransGen, Beijing, China) with corresponding primers on CFX96^®^ Real-Time PCR system (Bio-Rad, Hercules, California, USA), and *OsACTIN* genes was used as the internal control for normalization. A complete list of primers is included in Supplementary Table S4. The relative transcript levels of indicated genes were quantified by 2^−ΔΔCT^ method from the qRT-PCR data of three biological replicate experiments with three independent repeat (Schmittgen and Livak, 2008).

### Subcellular localization

The protein-coding sequences of *OsLTPL23* (without the termination codon) were cloned from the cDNA of 2-weeks old NIP seedlings, and inserted into the PMDC43 and the pSAT6-eYFP**-**N1 vector, respectively. Then, the plasma membrane marker AtPIP2A-mCherry (Cutler et al., 2000) or nucleus marker bZIP73-mCherry (Liu et al., 2019) were separately introduced into *N. benthamiana* epidermal cells with PMDC43-OsLTPL23 construct *via Agrobacterium tumefaciens* strain EH105-mediated transient transformation or infiltrated into rice protoplasts with pSAT6-OsLTPL23-eYFP-N1 construct using polyethylene glycol (PEG)-mediated transformation method, respectively (Chen et al., 2008). The fluorescence intensity of recombinant proteins was photographed at ZEISS LSM 710 NLO (Carl Zeiss, Oberkochen, Germany).

### Phylogenetic analysis

The primary sequences of OsLTPL23 were blasted and aligned to the plant homologues in NCBI and Phytozome database. A neighbour-joining phylogenetic tree between OsLTPL23 and its homologues was constructed by MEGA X with the Poisson correction model, pairwise deletion for gaps/missing data treatment and 1,000 of bootstrap replicates (1,000 replicates) (Kumar et al., 2016).

### Starch and soluble sugar content measurement

The dry rice seeds were successively ground into fine powder filtered with 100-, 200-, and 400-mesh sieves. The amount of starch and soluble sugar was separately quantified with 0.03 g and 0.2 g samples according to the manufacturer’s instructions (BC0705, BC0035; Solarbio, Beijing, China).

### Data analysis

All experimental data were performed with GraphPad Pism **8** software and statistical analyses among samples were compared using Student’s t-test at the 5% and 1% levels of probability.

## Results

### *OsLTPL23* may be associated with seed germination in rice

**FIGURE 1:**
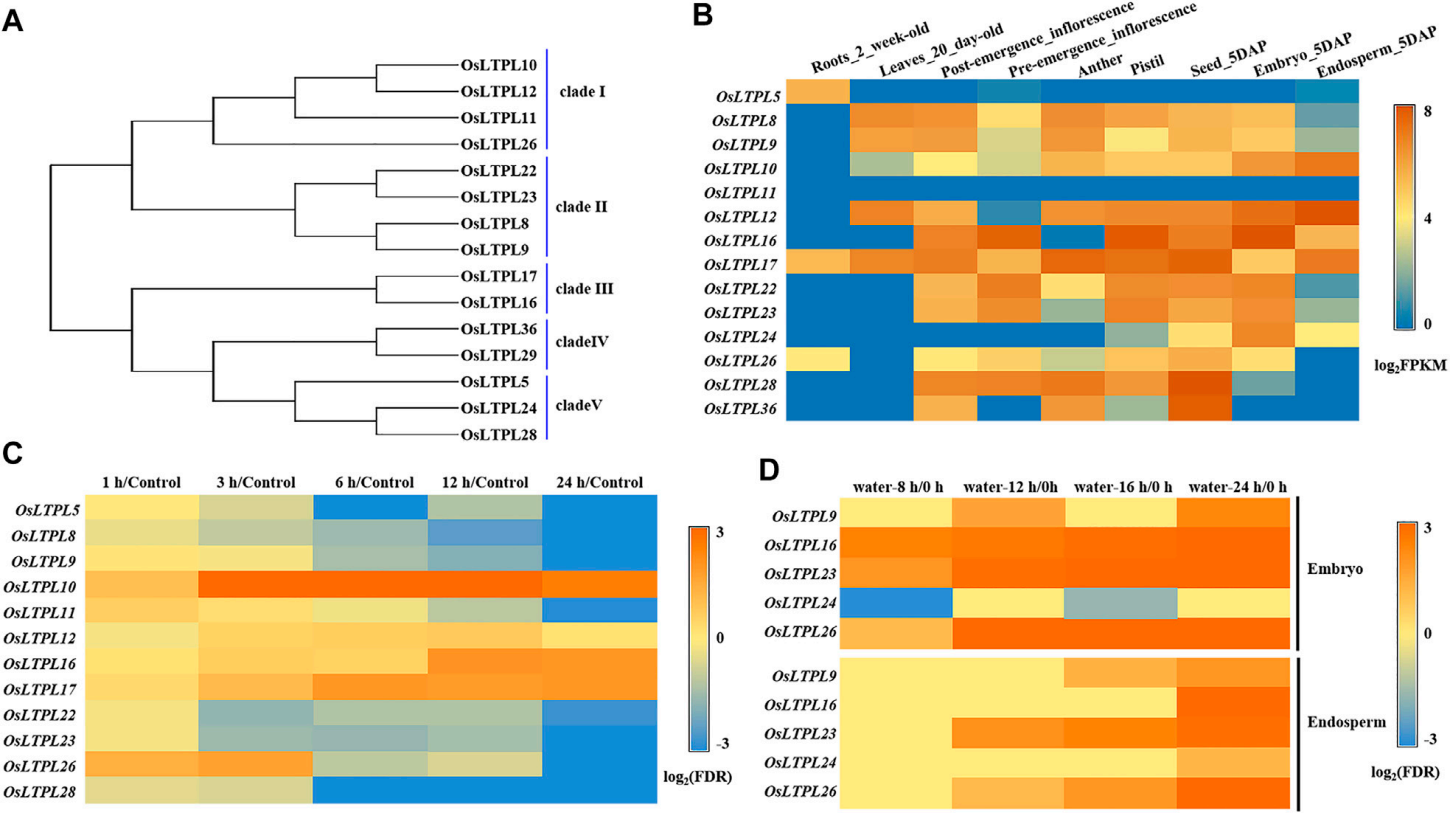
*OsLTPL23* is specific expressed in seed development and germination process of rice. **(A)** Phylogenetic tree of the reported rice nsLTP genes expressed in seed or embryo. **(B)** Tissue expression profiles of rice nsLTP genes from Plant Regulomics. **(C)** Transcription patterns of rice nsLTP genes response to ABA treatments. Blue and orange red boxes represent decreased and enriched transcript abundance, respectively. The gene expression level at 0 h after ABA treatment serves as control. **(D)** Transcription patterns of rice nsLTP genes in the imbibed embryo and endosperm under water treatment, respectively. Blue and orange red boxes represent down-regulated and up-regulated expression level, respectively.

To reach a better understanding of nsLTP-based regulation of seed vigor and identify the nsLTPs responsible for seed germination in rice, we functionally analyzed the reported rice nsLTP members with gene expression in seed or embryo (Wang et al., 2012). Through phylogenetic relationship, we found that a total of 15 nsLTP-encoding proteins can be classified into five clade (Figure 1A), which agrees with previous investigation (Wang et al., 2012). Followed the tissue expression profiles from the Plant Regulomics database, the transcripts of the nsLTP genes, *OsLTPL8*, *OsLTPL9*, *OsLTPL22* and *OsLTPL23*, in clade II were specifically enriched in the developing seed, with high expression in 5 days after pollination (DAP)-seed and -embryo and low accumulation in 5 DAP-endosperm, suggesting that this clade may be involved in carbohydrates accumulation during rice seed maturation (Figure 1B; Supplementary Table S1). The phytohormones ABA negatively regulates seed germination and maintains seed dormancy (Finkelstein et al., 2008; Rajjou et al., 2012; Penfield, 2017). To determine the candidate genes controlling seed germination, the responsive profiles of these nsLTP genes to ABA were downloaded from TENOR (https://tenor.dna.affrc.go.jp/) and investigated. The transcript abundance of *OsLTPL5*, *OsLTPL8*, *OsLTPL9*, *OsLTPL11*, *OsLTPL22*, *OsLTPL23*, *OsLTPL26* and *OsLTPL28* genes gradually decreased along ABA treatment, providing the possibility of *OsLTPL8*, *OsLTPL9*, *OsLTPL22* and *OsLTPL23* in regulating seed germination (Figure 1C; Supplementary Table S2). Further, we found that the transcription expression of three genes, *OsLTPL16*, *OsLTPL23* and *OsLTPL26,* are induced in imbibed seeds, which reinforces the reliability of *OsLTPL23* participating in seed germination (Figure 1D; Supplementary Table S3; Galland et al., 2014). Collectively, these data pointed to the idea that *OsLTPL23* is the most candidate related to seed vigor and seed germination in these rice nsLTP members.

### *OsLTPL23* encodes a non-specific lipid transfer protein

**FIGURE 2:**
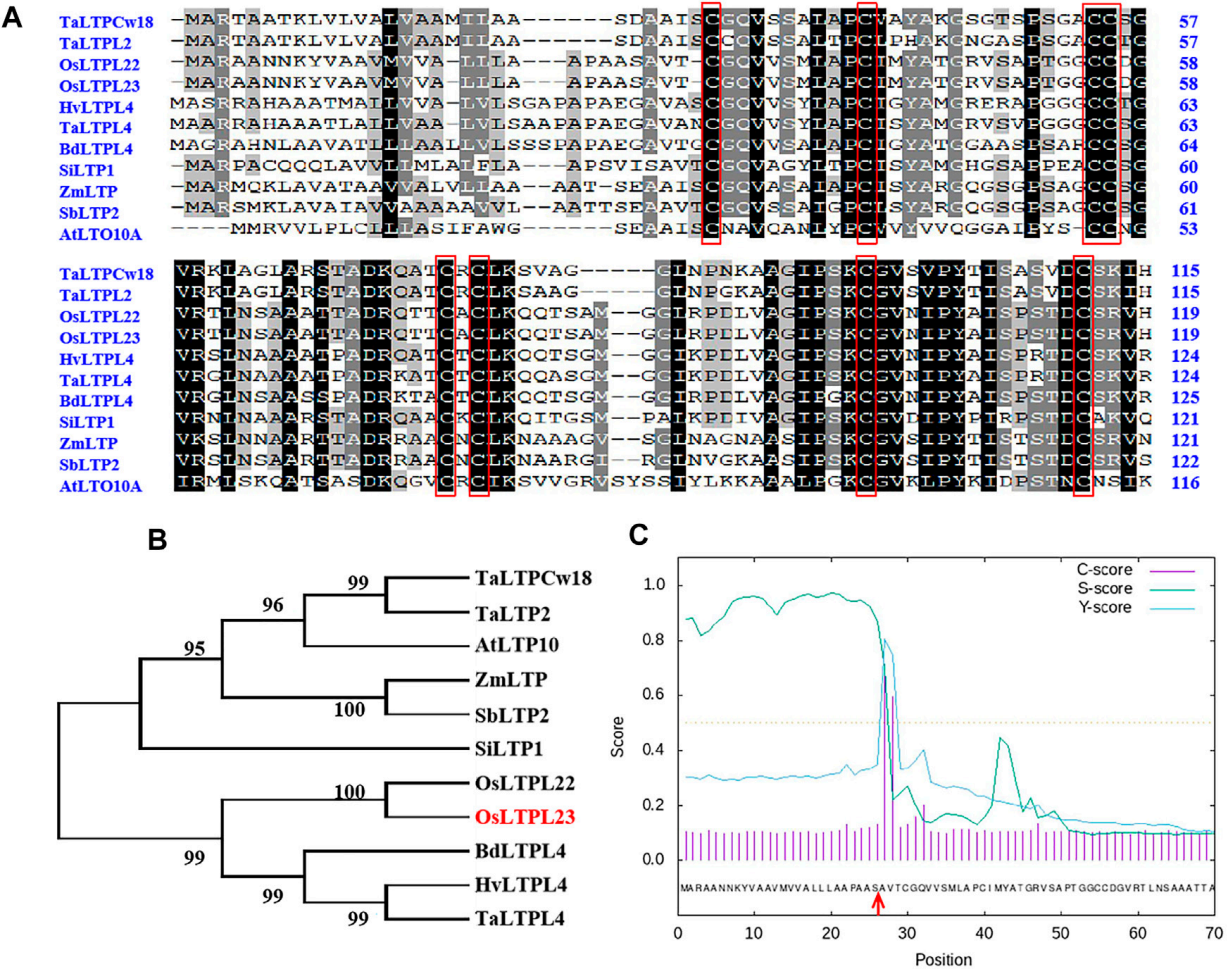
Sequence analysis of OsLTPL23 protein. **(A)** Primary sequence alignment of OsLTPL23 with homologues. Conserved eight-cysteine motif are represented by red rectangles. **(B)** Phylogenetic tree of OsLTPL23. Ta, *Triticum aestivum*; At, *Arabidopsis thaliana*; Zm, *Zea mays*; Sb, *Sorghum bicolor*; Si, *Setaria italica*; Os, *Oryza sativa*; Bd, *Brachypodium distachyon*; Hv, *Hordeum vulgare*. **(C)** Signal peptide prediction of OsLTPL23. The red arrows indicate the predicted signal peptide cleavage site using SignalP4.1 (https://services.healthtech.dtu.dk/service.php?SignalP-4.1).

**FIGURE 3:**
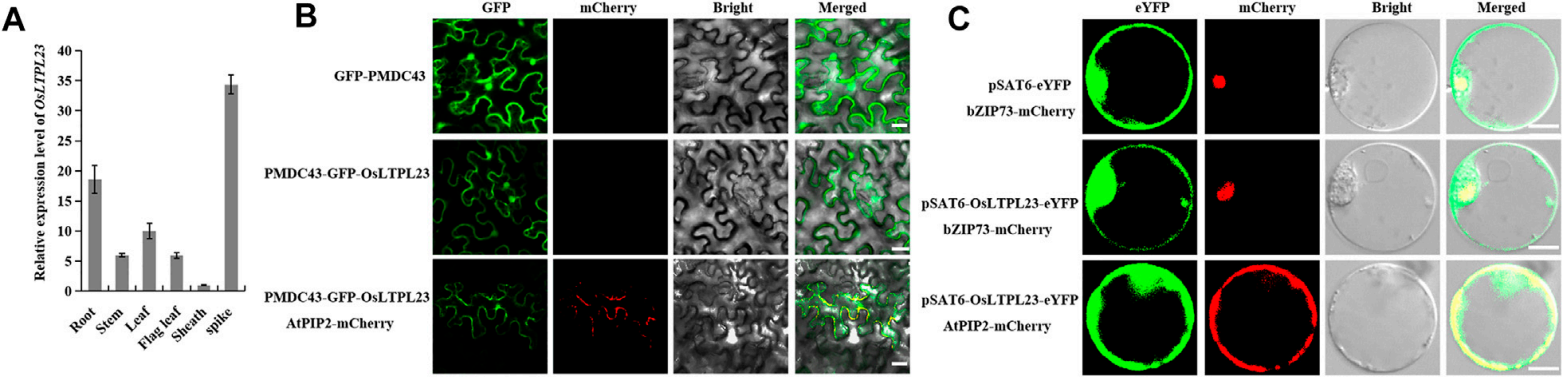
Tissue expression pattern and subcellular localization of *OsLTPL23*. **(A)** *OsLTPL23* transcription expression in various tissues of NIP using qRT-PCR. **(B)** Subcellular localization of GFP-OsLTPL23 fusion protein in *N. benthamiana* epidermal cells. Plasmids PMDC43 and PMDC43-OsLTPL23 were introduced into tobacco leaf cells by *Agrobacterium*-mediated transformation, respectively. Scale bars, 20 μm. **(C)** Subcellular localization of OsLTPL23-eYFP fusion protein in rice protoplast. Plasmids pSAT6-eYFP-N1 and pSAT6-OsLTPL23-eYFP-N1 were introduced into rice protoplast by PEG-mediated transformation, respectively. Scale bar, 10 μm *OsbZIP73* (*bZIP transcription factor*, nucleus marker, *Os09g0474000*); *AtPIP2A* (*plasma membrane intrinsic protein 2A*, *At3g53420*).

To ascertain the potential relationship between OsLTPL23 and seed germination, a phylogram of OsLTPL23 and its plant homologues was firstly generated through the amino acid (aa) sequence based neighbor-joining algorithm (Figures 2A, B). Homology analysis in 7 model plants displayed that the proteins high identity to OsLTPL23 are presented in gramineous C3 plants, including *Triticum aestivum*, *Hordeum vulgare*, *Brachypodium distachyon*. Unfortunately, there were no reports on the function of those proteins in seed germination. By the protein structure analysis, OsLTPL23 encodes a typical plant non-specific lipid transfer protein with 120 amino acids, including one 26 aa N-terminal hydrophobic signal peptide and the 8 CM in bifunctional inhibitor/plant lipid transfer protein/seed storage helical domain (Figure 2A, C). Subsequently, we isolated total RNA from six tissue samples of NIP at vegetative growth and reproductive growth stages to verify the tissue specific expression pattern of *OsLTPL23*. The qRT-PCR analysis indicated that the highest gene expression level of *OsLTPL23* is presented in rice spikes, followed by that in roots and leaves at five-leaf stage, and decreased in flag leaves, stem and sheathes at booting stage (Figure 3A). These results basically agreed with the data from the Plant Regulomics database, supporting the function of *OsLTPL23* in seed development.

Several investigations showed that the rice nsLTP family members specifically localized to the plasma membrane, cytoplasm and nucleus to regulate the cold tolerance, fertility, seed development and low-temperature germinability, respectively (Fujino and Sekiguchi, 2011; Li et al., 2020; Zhao et al., 2020; Chen et al., 2022). To detect the functional compartment of OsLTPL23 at subcellular level, we monitored the fluorescence intensity of OsLTPL23 recombinant protein in different transient expression systems. The fluorescence signal in *Nicotiana* *benthamiana* exhibited that the fusion protein GFP-OsLTPL23 clearly localizes to the nucleus and colocalizes with the plasma membrane marker AtPIP2A-mCherry (Figure 3B). To further determine the subcellular localization of OsLTPL23, pSAT6-eYFP, OsbZIP73-mCherry, AtPIP2A-mCherry and pSAT6-OsLTPL23-eYFP constructs were also introduced into the rice protoplast. Laser scanning confocal microscope showed that the recombinant protein OsLTPL23-eYFP separately colocalizes with the plasma membrane marker AtPIP2A-mCherry and nucleus marker OsbZIP73-mCherry in rice (Figure 3C), implying that *OsLTPL23* may be involved in transcriptional regulation, as well as plasma membrane biological function.

### The *osltpl23* mutants are created by genome editing

**FIGURE 4:**
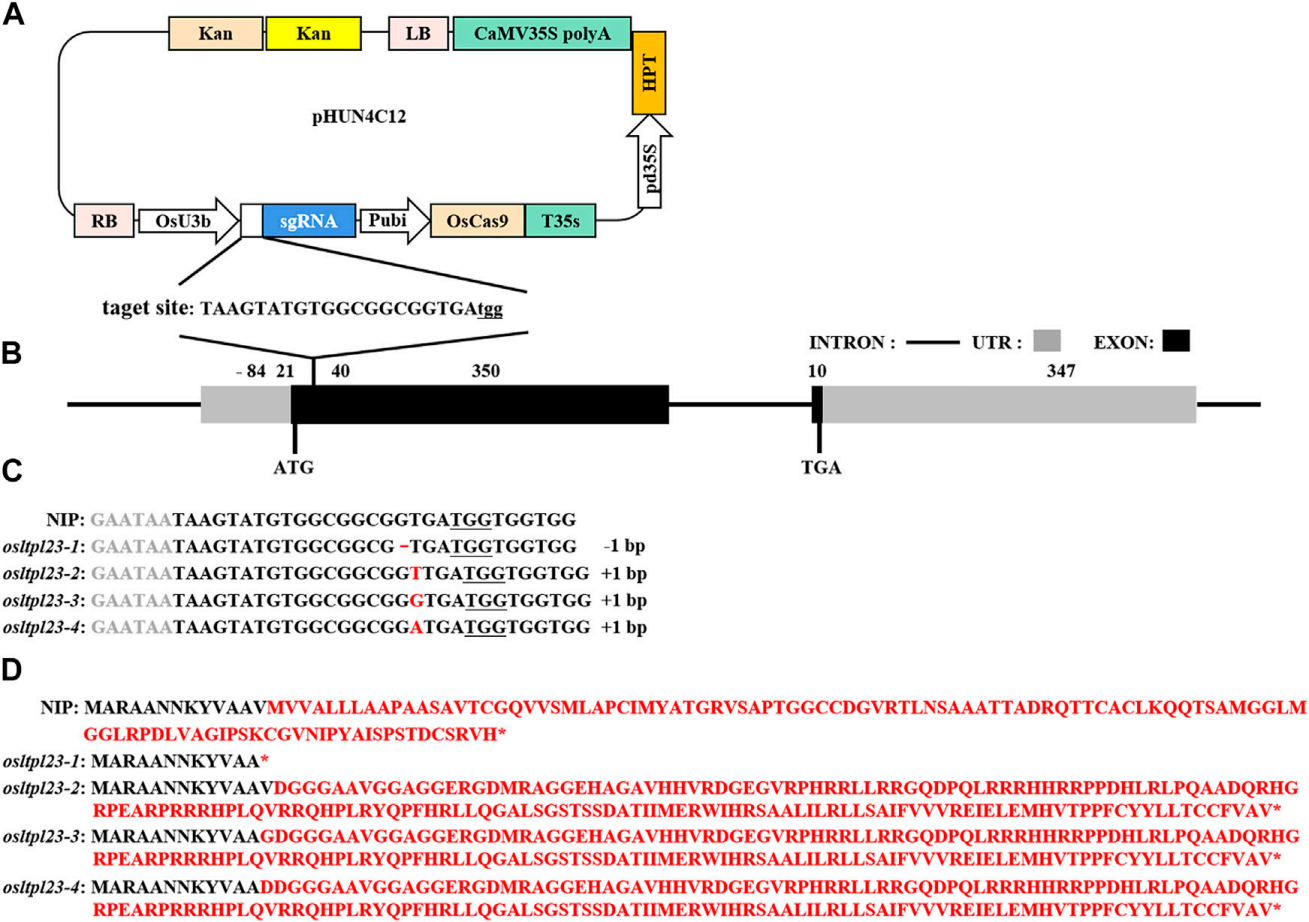
Cas9-mediated *OsLTPL23* gene mutation in rice. **(A)** Target site sequence and CRISPR/Cas9 vector structure. Capital letters indicates the sgRNA sequences, and three underlined lowercase letters represent the protospacer adjacent motif (PAM). The expression of *Cas9*, sgRNA and *hygromycin* (*HPT*) is separately driven by the maize ubiquitin promoter (Pubi), rice U3b promoter (OsU3b) and 35S promoters (pd35S). **(B)** Schematic of the *OsLTPL23* gene structure. Black rectangles, gray rectangles and black lines represent exon, untranslated region (UTR) and intron, respectively. **(C)** The gene editing events in the four rice homozygous mutants. The underlined nucleotide sequences are PAM. The red lines and capital letters represent deleted nucleotide and inserted nucleotide, respectively. **(D)** Predicted protein sequences encoded by mutational *OsLTPL23* in *osltpl23* mutants.

To explore the involvement of *OsLTPL23* gene in rice seed germination, the generation of *osltpl23* mutants in the NIP background was achieved through Cas9-induced gene editing (Figure 4A). Specifically, the target sequences were located at the position of 21–40 bp in the first exon of *OsLTPL23* (Figure 4B). After the sequencing of site-specific PCR products, 12 *osltpl23* mutants, including four homozygous and eight heterozygous mutations, were recovered from 21 T_0_ hygromycin-resistant transgenic plants (57.1%). To obtain more allele mutation types, the zygosity analysis of eight heterozygous mutants were carried out through the T vector sequencing of PCR products. Based on the results of zygosity analysis, 33.3%, 19.0% and 33.3% of the mutations were separately nucleotide insertions, deletions and substitutions, and others were characterized by the chimeric mutation (Figure 4C; Supplementary Figure S1).

To obtain the transgene-free plants, all the T_0_ mutants were self-pollinated, and we isolated two T-DNA-free homozygous lines from T_1_ generation of *osltpl23-1* and *osltpl23-2* mutants. As shown in Figures 4C, D, the *osltpl23-1* and *osltpl23-2* mutants separately harbored a 1 bp deletion and insertion between nucleotide 37 and 39 of the *OsLTPL23* coding region, which result in a truncated and recombinant variant of the OsLTPL23 respectively. Besides, we noticed that *osltpl23-3* and *osltpl23-4* mutants produce nearly identical protein variants with *osltpl23-2* mutant. Given no differentially expressed *OsLTPL23* in all the *osltpl23-2*, *osltpl23-3,* and *osltpl23-4* mutants (Supplementary Figure S2), the *osltpl23-1* and *osltpl23-2* mutants were subjected to further investigation.

### The rice mutants *osltpl23* displays slower and lower seed germination

**FIGURE 5:**
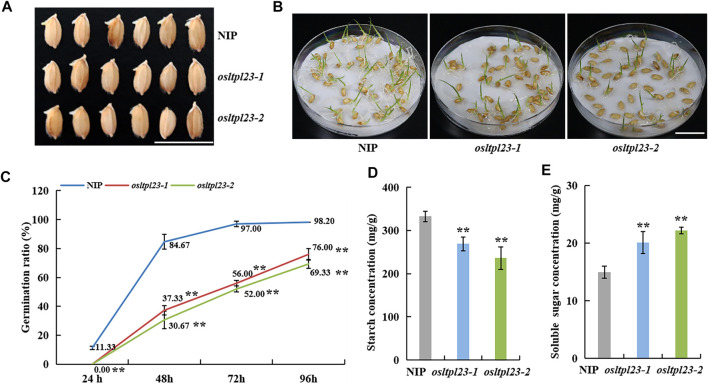
*OsLTPL23* positively regulates rice seed germination. **(A)** Phenotypes of germinated seeds of NIP, *osltpl23-1* and *osltpl23-2* plants after 3 days. Scale bar, 2 cm. **(B)** Phenotypes of germinated seeds of NIP, *osltpl23-1* and *osltpl23-2* plants after 6 days. Scale bar, 2 cm. **(C)** Germination percentage of wild-type NIP, *osltpl23-1* and *osltpl23-2* mutant plants. **(D)** Starch content determination of dry seeds in NIP, *osltpl23-1* and *osltpl23-2* plants. **(E)** Soluble sugar content determination of dry seeds in wild-type NIP, *osltpl23-1* and *osltpl23-2* plants. Values are the mean ±standard deviations of three biological replicates. Significance were generated by Student’s *t*-test with *p < .05 and **p < .01.

To validate the germination-responsiveness of *OsLTPL23*, seeds from wild type NIP, *osltpl23-1* and *osltpl23-2* mutants were harvested and stored. The seeds stored for 18 months post-harvest were soaked in distilled water to calculate the germination rate. The osltpl23 mutants supported distinctly slower germination before 24 hpi and lower germination percentage at 24 hpi to 96 hpi than NIP (Figures 5A–C). At 24 hpi, the germination percentage of NIP (11.83%) was clearly higher than that of *osltpl23* (0%). Subsequently, there were a sharply augmented difference between NIP and *osltpl23* mutants at 48 hpi and 72 hpi, which the germination percentages of NIP were 2.27- and 2.76-fold, and 1.73- and 1.86-fold that of *osltpl23-1* and *osltpl23-2* mutant, respectively. Eventually, the germination percentages of NIP and *osltpl23* mutants at 96 h reached 98.20%、76.00% and 69.33%, respectively (Figure 5C). These data indicated that the dysfunction of *OsLTPL23* delays and inhibits seed germination.

### *OsLTPL23* blocks the hydrolysis of starch into soluble sugar

In previous protein structure analysis, OsLTPL23 has a bifunctional inhibitor/plant lipid transfer protein/seed storage helical domain. Further functional domain analysis showed that it may act as trypsin-alpha amylase inhibitor (https://www.ebi.ac.uk/interpro/entry/pfam/PF00234/). In rice, alpha-amylas isozymes are critical to convert the stored starch into soluble sugar for nourishing the seedling establishment (Damaris et al., 2019). To validate the role of *OsLTPL23* in the conversion between starch and soluble sugar, we measured the starch and soluble sugar contents in the dry seeds of NIP, *osltpl23-1* and *osltpl23-2* mutants. The results uncovered that the starch contents of *osltpl23-1* and *osltpl23-2* mutants, with separately 268.90 mg/g and 236.17 mg/g, were significantly reduced compared with NIP (332.73 mg/g), whilst the soluble sugar contents were increased 34.11% and 48.19% relative to 14.98 mg/g in NIP (Figures 5D, E). There are reports that soluble sugar delays seed germination (Zhu et al., 2009), we speculated that the high soluble sugar contents may be a contributor responsible for the slower germination in *osltpl23* mutants.

### Endogenous ABA participates in *OsLTPL23*-mediated seed germination

**FIGURE 6:**
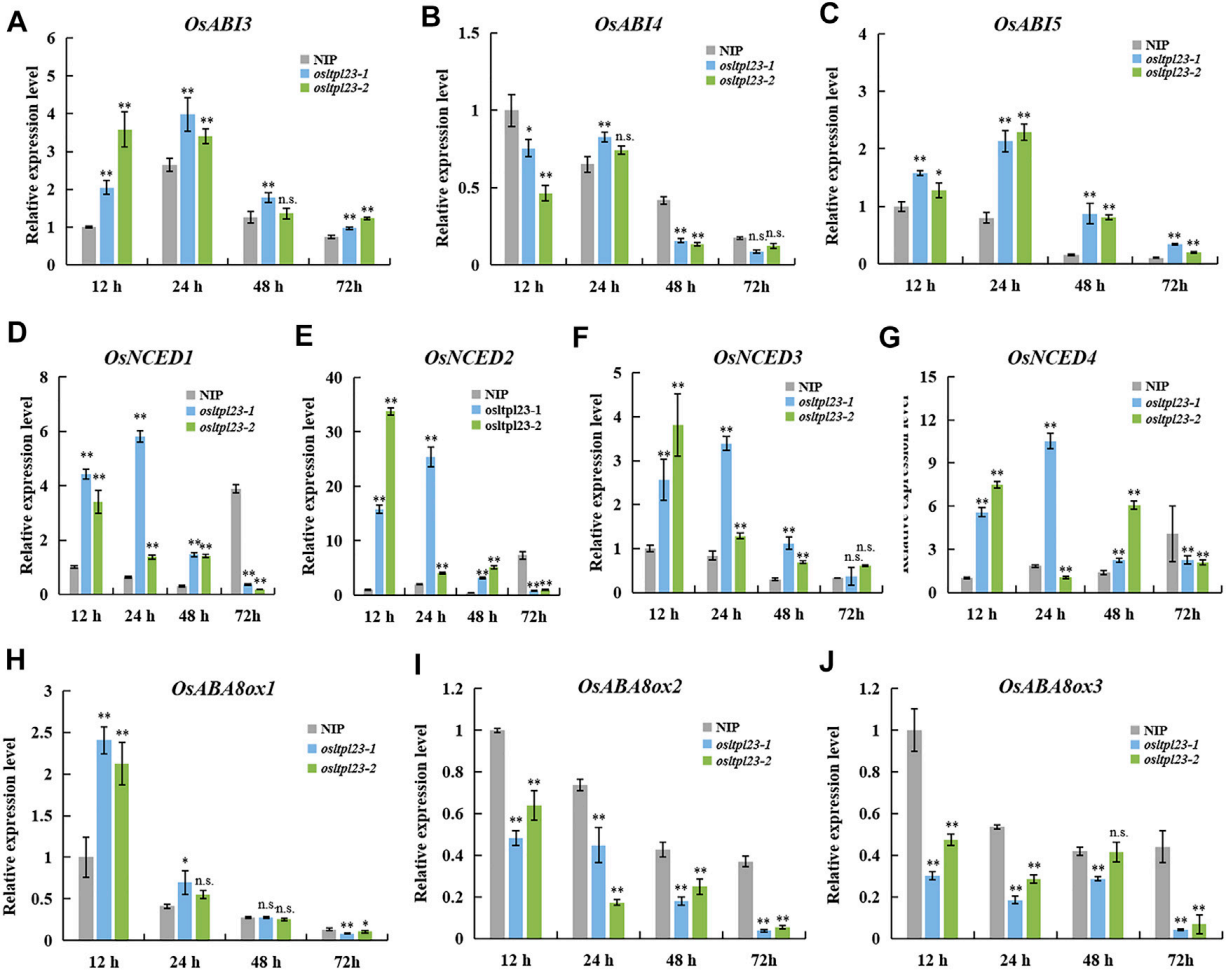
*OsLTPL23* alters the transcriptional expression of the genes in ABA-responsive, -biosynthesis and -metabolism pathway during seed germination. **(A–C)** The *OsABI3* **(A)**, *OsABI4* **(B)** and *OsABI5* **(C)** expression in germinated seeds among NIP, *osltpl23-1* and *osltpl23-2* plants. **(D–J)** The relative gene expression levels of ABA biosynthesis genes *OsNCED1* **(D)**, *OsNCED2* **(E)**, *OsNCED3* **(F)** and *OsNCED4* **(G)**, and ABA metabolism genes *OsABA8ox1* **(H)**, *OsABA8ox2* **(I)**, and *OsABA8ox3* **(J)** in germinated seeds among NIP, *osltpl23-1* and *osltpl23-2* plants. The gene expression levels of NIP at 12 hpi was used as control. Values are the mean ±standard deviations of three biological replicates. Significance were generated by Student’s t-test with *p < .05 and **p < .01.

ABA is a central player in controlling seed dormancy and germination (Finkelstein et al., 2008; Rajjou et al., 2012; Penfield, 2017). To investigate whether ABA is involved *OsLTPL23*-trigger seed germination reduction, we firstly checked the expression level of ABA-responsive genes *OsABI3*, *OsABI4* and *OsABI5*, which are major downstream components of ABA signalling pathway in seed dormancy and seed germination (Lopez-Molina et al., 2001; Liu et al., 2013; Luo et al., 2021; He et al., 2022). The transcripts of *OsABI3* and *OsABI5* in *osltpl23* mutants obviously accumulated compared with those of NIP within 72 hpi, whereas the relative *OsABI4* expression in *osltpl23-1* mutant were only higher than NIP at 24 hpi (Figures 6A–C). Pioneer report has demonstrated that the ABA content of *abi4* seeds was comparable with wild type after stratification (Shu et al., 2013). Therefore, we speculated that the amounts of ABA in *osltpl23* mutants may be higher than that in NIP.

To investigate whether endogenous ABA is increased in *osltpl23* mutants, the transcription levels of crucial ABA synthesis and metabolism genes (Figures 6D–J), including *OsNCEDs* (ABA biosynthesis) and *OsABA8oxs* (ABA degradation), were quantified among the imbibed seeds of NIP, *osltpl23-1* and *osltpl23-2* mutants. The gene transcription amounts of *OsNCED1*, *OsNCED2*, *OsNCED3*, and *OsNCED4* were clearly upregulated in *osltpl23* lines, compared with NIP within 12–48 hpi, whilst the increase trend of all the *OsNCEDs* were blocked at 72 hpi (Figures 6D–G). Concurrently, the mRNA amounts of *OsABA8ox2* and *OsABA8ox3* were markedly abolished in *osltpl23* mutants relative to those in NIP at 12–72 hpi, whereas the transcription expression of *OsABA8ox1* was gradually decreased within 3 days post imbibition (Figures 6H–J). Taken together, these data exposed that the disruption of *OsLTPL23* may promote the endogenous ABA accumulation, thus leading to lower germination rate of *osltpl23* mutants.

### The *osltpl23* mutants develop weakened seedlings

**FIGURE 7:**
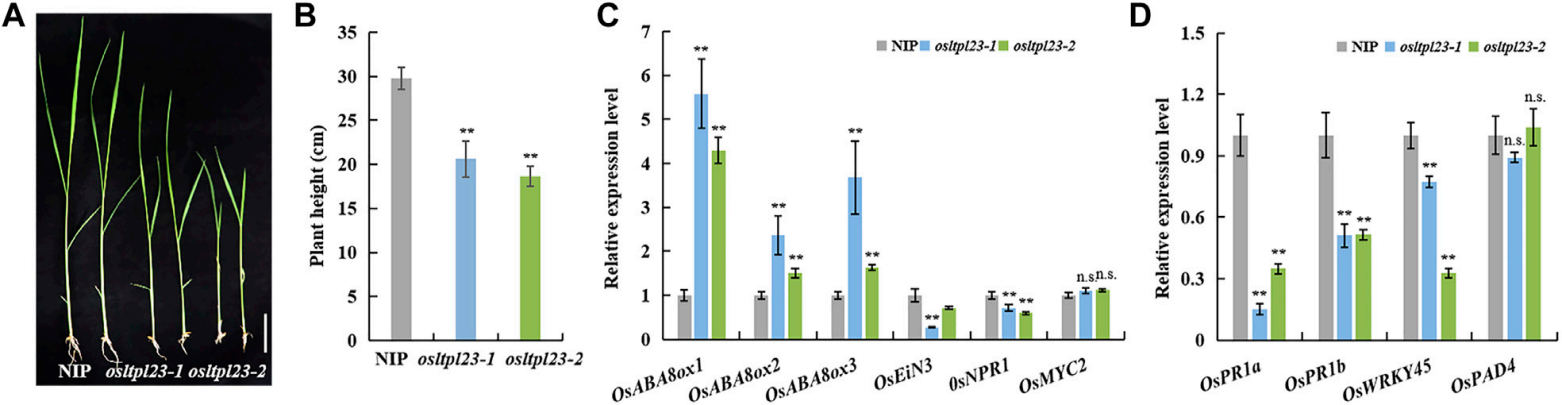
The *osltpl23* mutants have weakened seedling growth. **(A)** Growth potential of NIP, *osltpl23-1* and *osltpl23-2* mutants after 14 days. Scale bar, 4 cm. **(B)** The plant height of 14 days old seedlings in NIP, *osltpl23-1* and *osltpl23-2* mutants. Scale bar, 4 cm. **(C)** Responsive expression of ABA-, ethylene-, JA- and SA-related genes of 14 days old seedlings in NIP, *osltpl23-1* and *osltpl23-2* mutants. **(D)** Expression level of defense-responsive genes of 14 days old seedlings in NIP, *osltpl23-1* and *osltpl23-2* mutants. Values are the mean ±standard deviations of three biological replicates. Significance were generated by Student’s t-test with *p < .05 and **p < .01.

The transcript enrichment of *OsABI3*, *OsABI5*, *OsNCEDs*, and accumulative soluble sugar observed in *osltpl23* mutants during seed germination provide a indicative cue that *OsLTPL23* may regulate post-germination growth of rice. To verify the possibility, we observed the growth phenotype of 2-week old seedlings grown in greenhouse. Phenotypic investigation showed that the plants from *osltpl23-1* and *osltpl23-2* lines have significantly lower seedling height, with 30.74% less and 37.35% less, than the control plants, respectively (Figures 7A, B). To confirm whether the post-germination seedling growth inhibition of *osltpl23* lines is ABA-dependent, we detected the mRNA levels of plant stress hormone-related genes in 2-week old seedlings between mutants and NIP. The qRT-PCR data displayed that all three genes, *OsABA8ox1*, *OsABA8ox2* and *OsABA8ox3* in ABA metabolic pathway are evidently increased in *osltpl23* mutants relative to those of NIP, whereas ethylene signal transduction gene *OsEiN3,* salicylic acid signal pathway gene *OsNPR1,* and jasmonic acid-responsive transcription factor *OsMYC2* have abortive or minor transcript enrichment in *osltpl23* lines compared with those of NIP (Figure 7C). These data jointly pinpoint the idea that the ABA is the major limiting factor for early seedling growth in *osltpl23* mutants. Meanwhile, we also checked the transcription levels of defence-related marker genes *OsPR1a*, *OsPR1b*, *OsWRKY45* and *OsPAD4* in all plants. All these biotic stress-responsive genes in the *osltpl23* lines exhibited lower gene expression level compared with those in NIP (Figure 7D). All these results indicated that *OsLTPL23* may positively regulate the rice early seedling growth.

## Discussion

The molecular dictation from a lower ABA/GA ratio in seeds determines the stage transition from dormancy to germination (Jacobsen et al., 2002; Piskurewicz et al., 2008; Shu et al., 2016; Yang et al., 2020). Sucrose is a rapidly consumed agent for growing embryonic axis in germinated seeds, which products, soluble sugar, is another factor influencing the germination process (Dekkers et al., 2004; Gibson, 2005; Zhu et al., 2009; Li et al., 2017; Matsukura et al., 2020). The effect and interplay between ABA and soluble sugar had been wildly investigated in the germinated seed (Dekkers et al., 2004; Zhu et al., 2009; Shu et al., 2013; Wang et al., 2021; Xue et al., 2021). In this present study, we provided evidences to depict the roles of lipid transfer protein OsLTPL23 in endogenous carbohydrates phase transition and seed germination. The abnormal starch-to-sugar conversion of the dry seed may lead to the delayed germination initiation in *osltpl23* mutants, and endogenous ABA homeostasis is involved in *OsLTPL2* *3-*controlled seed germination vigor. These results offered a new viewpoint regarding how nsLTPs act in the metabolism of carbohydrates and ABA during seed germination.

Plant nsLTPs are a class of small, secreted proteins, which localize in the cell wall (Thoma et al., 1993), plasma membrane (Li et al., 2020; Zhao et al., 2020; Chen et al., 2022), cytoplasm (Fujino et al., 2008; Li et al., 2020) and nucleus (Fujino et al., 2008). Accumulated evidences have shown that nsLTPs play important roles in wax assembly (Hollenbach et al., 1997), cell wall extension (Nieuwland et al., 2005), anther and pollen development (Chae et al., 2009; Zhang et al., 2010), seed development and quality (Wang et al., 2015) and plant-pathogen interaction (Segura et al., 1993; Molina and Garcia-Olmedo, 1997; Park et al., 2002; Gomes et al., 2003; Sarowar et al., 2009; Ahmed et al., 2017). Here, we identified a plasma membrane and nucleus colocalized nsLTP, OsLTPL23, which is distinct from starch-independent *OsLTPL36* and ABA-dependent *OsTPP1* in seed germination (Wang et al., 2015; Wang et al., 2021). We speculated that the putative alpha-amylase inhibitor activity and nucleus enrichment together issue in the phenotype of *osltpl23* mutants, especially the nucleus localization representing the manipuility of ABA-related gene expression.

The stored starch in the endosperm is the major energy source during seed germination and seedling establishment, while the inhibitory effect on seed germination and post-germination seedling growth is largely dependent on the increase of ABA concentration (Damaris et al., 2019; Chen et al., 2020). In imbibed seed, the glucose, one hydrolysate of starch, treatment delays the germination process by alleviating endogenous ABA degradation, exogenous ABA, in turn, controls endogenous glucose partition to inhibit germination (Dekkers et al., 2004; Zhu et al., 2009; Xue et al., 2021). Initially, *OsLTPL23* was identified as candidate positive regulator responsible for seed germination, with induced transcription in embryo and endosperm. Paradoxically, our carbohydrates determination result suggested that OsLTPL23 has alpha-amylase inhibitor activity in dry seeds, which must destroy the energy source of seed germination, seedling establishment and seedling growth through restraining starch-to-sugars conversion. There are two possible explanation for this discordance. One is that the microarray-based gene expression cannot reflect the natural transcription state of *OsLTPL23*. The other is that *OsLTPL23* may not an key contributor in seed germination and post-germination seedling development. Here, we preferred the former, because *osltpl23* mutants shows increased soluble sugar concentration in the dry seeds and the transcription feature of elevated ABA content in germinated seeds and early seedling growth. In the future, the molecular mechanism of endogenous ABA and soluble sugar interaction on seed germination should be emphasized. Overall, *osltpl23* mutants provide an important resource to survey the seed germination, post-germination growth and seed development, as well as high vigor seed production in rice.

## Data Availability

The original contributions presented in the study are included in the article/Supplementary Material, further inquiries can be directed to the corresponding author.

## Supplementary material

SUPPLEMENTARY FIGURE S1 Nucleotide sequences at the target site in eight heterozygous mutants. The recovered mutated alleles are shown below the NIP sequence. The target site nucleotides are indicated with black capital letters. The PAM site is underlined. The red dashes indicate deleted nucleotides. The red capital letters indicate inserted nucleotides.

SUPPLEMENTARY FIGURE S2 OsLTPL23 expression detection in osltpl23 mutants. Each column presents the means±standard deviations of three biological replicates. Values are the mean±standard deviations of three biological replicates. Significance were generated by Student’s t-test with **p* < .05 and ***p* < .01

## References

[B1] Ahmed S. M. Liu P. Xue Q. Ji C. Qi T. Guo J. (2017). TaDIR1-2, a wheat ortholog of lipid transfer protein AtDIR1 contributes to negative regulation of wheat resistance against *Puccinia striiformis f.* sp. *tritici* . Front. Plant Sci. 8, 521. 10.3389/fpls.2017.00521 28443114 PMC5387106

[B2] Arroyo A. Bossi F. Finkelstein R. R. Leon P. (2003). Three genes that affect sugar sensing (*abscisic acid insensitive 4, abscisic acid insensitive 5*, and *constitutive triple response 1*) are differentially regulated by glucose in *Arabidopsis* . Plant Physiol. 133, 231–242. 10.1104/pp.103.021089 12970489 PMC196600

[B3] Bossi F. Cordoba E. Dupre P. Mendoza M. S. Roman C. S. Leon P. (2009). The *Arabidopsis ABA-INSENSITIVE* (*ABI) 4* factor acts as a central transcription activator of the expression of its own gene, and for the induction of *ABI5* and *SBE2.2* genes during sugar signaling. Plant J. 59, 359–374. 10.1111/j.1365-313X.2009.03877.x 19392689

[B4] Chae K. Kieslich C. A. Morikis D. Kim S. C. Lord E. M. (2009). A gain-of-function mutation of *Arabidopsis* lipid transfer protein 5 disturbs pollen tube tip growth and fertilization. Plant Cell 21, 3902–3914. 10.1105/tpc.109.070854 20044438 PMC2814499

[B5] Chen H. M. Zou Y. Shang Y. L. Lin H. Q. Wang Y. J. Cai R. (2008). Firefly luciferase complementation imaging assay for protein-protein interactions in plants. Plant Physiol. 146, 368–376. 10.1104/pp.107.111740 18065554 PMC2245818

[B6] Chen K. Li G. J. Bressan R. A. Song C. P. Zhu J. K. Zhao Y. (2020). Abscisic acid dynamics, signaling, and functions in plants. J. Integr. Plant Biol. 62, 25–54. 10.1111/jipb.12899 31850654

[B7] Chen L. B. Ji C. H. Zhou D. G. Gou X. Tang J. N. Jiang Y. J. (2022). *OsLTP47* may function in a lipid transfer relay essential for pollen wall development in rice. J. Genet. Genomics 49, 481–491. 10.1016/j.jgg.2022.03.003 35331929

[B9] Cutler S. R. Ehrhardt D. W. Griffitts J. S. Somerville C. R. (2000). Random GFP::DNA fusions enable visualization of subcellular structures in cells of *Arabidopsis* at a high frequency. Proc. Natl. Acad. Sci. USA. 97, 3718–3723. 10.1073/pnas.97.7.3718 10737809 PMC16306

[B10] Damaris R. N. Lin Z. Y. Yang P. F. He D. L. (2019). The rice alpha-amylase, conserved regulator of seed maturation and germination. Int. J. Mol. Sci. 20, 450–466. 10.3390/ijms20020450 30669630 PMC6359163

[B11] Dekkers B. J. Schuurmans J. A. Smeekens S. C. (2004). Glucose delays seed germination in *Arabidopsis thaliana* . Planta 218, 579–588. 10.1007/s00425-003-1154-9 14648119

[B12] Deng W. J. Li R. Q. Xu Y. W. Mao R. Y. Chen S. F. Chen L. B. (2020). A lipid transfer protein variant with a mutant eight-cysteine motif causes photoperiod- and thermo-sensitive dwarfism in rice. J. Exp. Bot. 71, 1294–1305. 10.1093/jxb/erz500 31701134 PMC7031082

[B13] Ding Z. J. Yan J. Y. Li G. X. Wu Z. C. Zhang S. Q. Zheng S. J. (2014). *WRKY41* controls *Arabidopsis* seed dormancy via direct regulation of *ABI3* transcript levels not downstream of ABA. Plant J. 79, 810–823. 10.1111/tpj.12597 24946881

[B14] Finch-Savage W. E. Bassel G. W. (2016). Seed vigour and crop establishment: Extending performance beyond adaptation. J. Exp. Bot. 67, 567–591. 10.1093/jxb/erv490 26585226

[B15] Finkelstein R. Reeves W. Ariizumi T. Steber C. (2008). Molecular aspects of seed dormancy. Annu. Rev. Plant Biol. 59, 387–415. 10.1146/annurev.arplant.59.032607.092740 18257711

[B16] Fleury C. Gracy J. Gautier M. F. Pons J. L. Dufayard J. F. Labesse G. (2019). Comprehensive classification of the plant non-specific lipid transfer protein superfamily towards its sequence-structure-function analysis. Peer J. 7, e7504. 10.7717/peerj.7504 31428542 PMC6698131

[B17] Foolad M. R. Subbiah P. Zhang L. (2007). Common QTL affect the rate of tomato seed germination under different stress and nonstress conditions. Int. J. Plant Genom. 97386, 97386. 10.1155/2007/97386 PMC2246063 18317505

[B18] Fujino K. Sekiguchi H. Matsuda Y. Sugimoto K. Ono K. Yano M. (2008). Molecular identification of a major quantitative trait locus, *qLTG3-1*, controlling low-temperature germinability in rice. Proc. Natl. Acad. Sci. USA. 105, 12623–12628. 10.1073/pnas.0805303105 18719107 PMC2527961

[B19] Fujino K. Sekiguchi H. (2011). Origins of functional nucleotide polymorphisms in a major quantitative trait locus, *qLTG3-1*, controlling low-temperature germinability in rice. Plant Mol. Biol. 75, 1–10. 10.1007/s11103-010-9697-1 20960223

[B20] Galland M. Boutet-Mercey S. Lounifi I. Godin B. Balzergue S. Grandjean O. (2014). Compartmentation and dynamics of flavone metabolism in dry and germinated rice seeds. Plant Cell Physiol. 55, 1646–1659. 10.1093/pcp/pcu095 25008975

[B21] Gibson S. I. (2005). Control of plant development and gene expression by sugar signaling. Curr. Opin. Plant Biol. 8, 93–102. 10.1016/j.pbi.2004.11.003 15653406

[B22] Gomes E. Sagot E. Gaillard C. Laquitaine L. Poinssot B. Sanejouand Y. H. (2003). Nonspecific lipid-transfer protein genes expression in grape (*Vitis* sp.) cells in response to fungal elicitor treatments. Mol. Plant Microbe Interact. 16, 456–464. 10.1094/MPMI.2003.16.5.456 12744517

[B23] Guo C. K. Ge X. C. Ma H. (2013). The rice *OsDIL* gene plays a role in drought tolerance at vegetative and reproductive stages. Plant Mol. Biol. 82, 239–253. 10.1007/s11103-013-0057-9 23686450

[B68] He Y. Chen W. T. Tan J. H. Luo X. X. Zhou T. J. Gong X. T. (2022). Rice CENTRORADIALIS 2 regulates seed germination and salt tolerance *via* ABA-mediated pathway. Theor Appl Genet 35, 4245–4259. 10.1007/s00122-022-04215-8 36181524

[B24] Hollenbach B. Schreiber L. Hartung W. Dietz K. J. (1997). Cadmium leads to stimulated expression of the lipid transfer protein genes in barley: Implications for the involvement of lipid transfer proteins in wax assembly. Planta 203, 9–19. 10.1007/s00050159 9299788

[B25] Jacobsen J. V. Pearce D. W. Poole A. T. Pharis R. P. Mander L. N. (2002). Abscisic acid, phaseic acid and gibberellin contents associated with dormancy and germination in barley. Physiol. Plant 115, 428–441. 10.1034/j.1399-3054.2002.1150313.x 12081536

[B26] Kader J. C. (1996). Lipid-transfer proteins in plants. Annu. Rev. Plant Physiology Plant Mol. Biol. 47, 627–654. 10.1146/annurev.arplant.47.1.627 15012303

[B27] Kumar S. Stecher G. Tamura K. (2016). MEGA7: Molecular evolutionary genetics analysis version 7.0 for bigger datasets. Mol. Biol. Evol. 33, 1870–1874. 10.1093/molbev/msw054 27004904 PMC8210823

[B28] Leprince O. Pellizzaro A. Berriri S. Buitink J. (2017). Late seed maturation: Drying without dying. J. Exp. Bot. 68, 827–841. 10.1093/jxb/erw363 28391329

[B29] Li H. J. Kim Y. J. Yang L. Liu Z. Zhang J. Shi H. T. (2020). Grass-specific *EPAD1* is essential for pollen exine patterning in rice. Plant Cell 32, 3961–3977. 10.1105/tpc.20.00551 33093144 PMC7721331

[B30] Li P. Zhou H. Shi X. L. Yu B. Zhou Y. Chen S. (2014a). The *ABI4*-induced *Arabidopsis ANAC060* transcription factor attenuates ABA signaling and renders seedlings sugar insensitive when present in the nucleus. PLoS Genet. 10, e1004213. 10.1371/journal.pgen.1004213 24625790 PMC3953025

[B31] Li R. Q. Xia J. X. Xu Y. W. Zhao X. C. Liu Y. G. Chen Y. L. (2014b). Characterization and genetic mapping of a *Photoperiod-sensitive dwarf 1* locus in rice (*Oryza sativa* L.). Theor. Appl. Genet. 127, 241–250. 10.1007/s00122-013-2213-7 24158250

[B32] Li T. Zhang Y. Wang D. Liu Y. Dirk L. M. Goodman J. (2017). Regulation of seed vigor by manipulation of raffinose family oligosaccharides in maize and *Arabidopsis thaliana* . Mol. Plant 10, 1540–1555. 10.1016/j.molp.2017.10.014 29122666

[B33] Liu C. T. Schlappi M. R. Mao B. G. Wang W. Wang A. J. Chu C. C. (2019). The *bZIP73* transcription factor controls rice cold tolerance at the reproductive stage. Plant Biotechnol. J. 17, 1834–1849. 10.1111/pbi.13104 30811812 PMC6686130

[B34] Liu X. D. Zhang H. Zhao Y. Feng Z. Y. Li Q. Yang H. Q. (2013). Auxin controls seed dormancy through stimulation of abscisic acid signaling by inducing *ARF* mediated *ABI3* activation in *Arabidopsis* . Proc. Natl. Acad. Sci. USA. 110, 15485–15490. 10.1073/pnas.1304651110 23986496 PMC3780901

[B35] Lopez-Molina L. Mongrand S. Chua N. H. (2001). A post germination developmental arrest checkpoint is mediated by abscisic acid and requires the *ABI5* transcription factor in *Arabidopsis* . Proc. Natl. Acad. Sci. USA. 98, 4782–4787. 10.1073/pnas.081594298 11287670 PMC31911

[B36] Luo X. F. Dai Y. J. Zheng C. Yang Y. Z. Chen W. Wang Q. C. (2021). The ABI4-RbohD/VTC2 regulatory module promotes reactive oxygen species (ROS) accumulation to decrease seed germination under salinity stress. New Phytol. 229, 950–962. 10.1111/nph.16921 32916762

[B37] Matsukura C. Saitoh T. Hirose T. Ohsugi R. Perata P. Yamaguchi J. (2000). Sugar uptake and transport in rice embryo. expression of companion cell-specific sucrose transporter (*OsSUT1*) induced by sugar and light. Plant Physiol. 124, 85–93. 10.1104/pp.124.1.85 10982424 PMC59124

[B38] Meng C. Yan Y. Liu Z. Chen L. Zhang Y. Li X. (2018). Systematic analysis of cotton non-specific lipid transfer protein family revealed a special group that is involved in fiber elongation. Front. Plant Sci. 9, 1285–1301. 10.3389/fpls.2018.01285 30283464 PMC6156462

[B39] Molina A. Garcia-Olmedo F. (1997). Enhanced tolerance to bacterial pathogens caused by the transgenic expression of barley lipid transfer protein LTP2. Plant J. 12, 669–675. 10.1046/j.1365-313x.1997.00669.x 9351251

[B40] Nieuwland J. Feron R. Huisman B. A. Fasolino A. Hilbers C. W. Derksen J. (2005). Lipid transfer proteins enhance cell wall extension in tobacco. Plant Cell 17, 2009–2019. 10.1105/tpc.105.032094 15937228 PMC1167548

[B41] Nishimura A. Aichi I. Matsuoka M. (2006). A protocol for Agrobacterium-mediated transformation in rice. Nat. Protoc. 1, 2796–2802. 10.1038/nprot.2006.469 17406537

[B42] Park C. J. Shin R. Park J. M. Lee G. J. You J. S. Paek K. H. (2002). Induction of pepper cDNA encoding a lipid transfer protein during the resistance response to tobacco mosaic virus. Plant Mol. Biol. 48, 243–254. 10.1023/a:1013383329361 11855726

[B43] Penfield S. (2017). Seed dormancy and germination. Curr. Biol. 27, R874–R878. 10.1016/j.cub.2017.05.050 28898656

[B44] Piskurewicz U. Jikumaru Y. Kinoshita N. Nambara E. Kamiya Y. Lopez-Molina L. (2008). The gibberellic acid signaling repressor RGL2 inhibits *Arabidopsis* seed germination by stimulating abscisic acid synthesis and ABI5 activity. Plant Cell 20, 2729–2745. 10.1105/tpc.108.061515 18941053 PMC2590721

[B45] Preston J. Tatematsu K. Kanno Y. Hobo T. Kimura M. Jikumaru Y. (2009). Temporal expression patterns of hormone metabolism genes during imbibition of *Arabidopsis thaliana* seeds: A comparative study on dormant and non-dormant accessions. Plant Cell Physiol. 50, 1786–1800. 10.1093/pcp/pcp121 19713425

[B46] Rajjou L. Duval M. Gallardo K. Catusse J. Bally J. Job C. (2012). Seed germination and vigor. Annu. Rev. Plant Biol. 63, 507–533. 10.1146/annurev-arplant-042811-105550 22136565

[B47] Salminen T. A. Blomqvist K. Edqvist J. (2016). Lipid transfer proteins: Classification, nomenclature, structure, and function. Planta 244, 971–997. 10.1007/s00425-016-2585-4 27562524 PMC5052319

[B48] Sarowar S. Kim Y. J. Kim K. D. Hwang B. K. Ok S. H. Shin J. S. (2009). Overexpression of lipid transfer protein (LTP) genes enhances resistance to plant pathogens and LTP functions in long‐distance systemic signaling in tobacco. Plant Cell Rep. 28, 419–427. 10.1007/s00299-008-0653-3 19089429

[B49] Schmittgen T. D. Livak K. J. (2008). Analyzing real-time PCR data by the comparative C(T) method. Nat. Protoc. 3, 1101–1108. 10.1038/nprot.2008.73 18546601

[B50] Segura A. Moreno M. Garcia-Olmedo F. (1993). Purification and antipathogenic activity of lipid transfer proteins (LTPs) from the leaves of Arabidopsis and spinach.Arabiopsis and spinach. FEBS Lett. 332, 243–246. 10.1016/0014-5793(93)80641-7 8405465

[B51] Shu K. Liu X. D. Xie Q. He Z. H. (2016). Two faces of one seed: Hormonal regulation of dormancy and germination. Mol. Plant 9, 34–45. 10.1016/j.molp.2015.08.010 26343970

[B52] Shu K. Zhang H. W. Wang S. F. Chen M. L. Wu Y. R. Tang S. Y. (2013). *ABI4* regulates primary seed dormancy by regulating the biogenesis of abscisic acid and gibberellins in *Arabidopsis* . PLoS Genet. 9, e1003577. 10.1371/journal.pgen.1003577 23818868 PMC3688486

[B53] Sun Q. Wang J. H. Sun B. Q. (2007). Advances on seed vigor physiological and genetic mechanisms. Agric. Sci. China 6, 1060–1066. 10.1016/S1671-2927(07)60147-3

[B69] Thoma S. Kaneko Y. Somerville C. (1993). A non-specific lipid transfer protein from Arabidopsis is a cell wall protein. Plant J 3, 427–436. 10.1046/j.1365-313x.1993.t01-25-00999.x 8220451

[B54] Tao Y. Zou T. Zhang X. Liu R. Chen H. Yuan G. Q. (2020). Secretory lipid transfer protein OsLTPL94 acts as a target of *EAT1* and is required for rice pollen wall development. Plant J. 108, 358–377. 10.1111/tpj.15443 34314535

[B55] Wang G. Q. Li X. Z. Ye N. H. Huang M. K. Feng L. Li H. X. (2021). *OsTPP1* regulates seed germination through the crosstalk with abscisic acid in rice. New Phytol. 230, 1925–1939. 10.1111/nph.17300 33629374

[B56] Wang H. W. Hwang S. G. Karuppanapandian T. Liu A. Kim W. Jang C. S. (2012). Insight into the molecular evolution of non-specific lipid transfer proteins via comparative analysis between rice and sorghum. DNA Res. 19, 179–194. 10.1093/dnares/dss003 22368182 PMC3325081

[B58] Wang X. Wei Z. Lu Z. H. Ouyang Y. D. Chol S. O. Yao J. L. (2015). A lipid transfer protein, OsLTPL36, is essential for seed development and seed quality in rice. Plant Sci. 239, 200–208. 10.1016/j.plantsci.2015.07.016 26398804

[B59] Wang Z. F. Wang J. F. Bao Y. M. Wang F. H. Zhang H. S. (2010). Quantitative trait loci analysis for rice seed vigor during the germination stage. J. Zhejiang Univ. Sci. B 11, 958–964. 10.1631/jzus.B1000238 21121075 PMC2997405

[B60] Xu R. F. Li H. Qin R. Y. Wang L. Li L. Wei P. (2014). Gene targeting using the *Agrobacterium* tumefaciens-mediated CRISPR-Cas system in rice. Rice 7, 5–8. 10.1186/s12284-014-0005-6 24920971 PMC4052633

[B61] Xue X. Yu Y. C. Wu Y. Xue H. Chen L. Q. (2021). Locally restricted glucose availability in the embryonic hypocotyl determines seed germination under abscisic acid treatment. New Phytol. 231, 1832–1844. 10.1111/nph.17513 34032290

[B62] Yang L. W. Liu S. R. Lin R. C. (2020). The role of light in regulating seed dormancy and germination. J. Integr. Plant Biol. 62, 1310–1326. 10.1111/jipb.13001 32729981

[B63] Zhang D. S. Liang W. Q. Yin C. S. Zong J. Gu F. W. Zhang D. B. (2010). *OsC6*, encoding a lipid transfer protein, is required for postmeiotic anther development in rice. Plant Physiol. 154, 149–162. 10.1104/pp.110.158865 20610705 PMC2938136

[B65] Zhao J. Wang S. Qin J. Sun C. Liu F. (2020). The lipid transfer protein OsLTPL159 is involved in cold tolerance at the early seedling stage in rice. Plant Biotechnol. J. 18, 756–769. 10.1111/pbi.13243 31469486 PMC7004919

[B66] Zhao M. Zhang H. X. Yan H. Qiu L. Baskin C. C. (2018). Mobilization and role of starch, protein, and fat reserves during seed germination of six wild grassland species. Front. Plant Sci. 9, 234. 10.3389/fpls.2018.00234 29535748 PMC5835038

[B67] Zhu G. H. Ye N. H. Zhang J. (2009). Glucose-induced delay of seed germination in rice is mediated by the suppression of ABA catabolism rather than an enhancement of ABA biosynthesis. Plant Cell Physiol. 50, 644–651. 10.1093/pcp/pcp022 19208695

