# Supplementary figures and images for: Rice lipid transfer protein, OsLTPL23, controls seed germination by regulating starch-sugar conversion and ABA homeostasis
Source: Front Genet. 2023 Jan 16;14:1111318. doi: 10.3389/fgene.2023.1111318 (PMC9885049; doi:10.3389/fgene.2023.1111318)

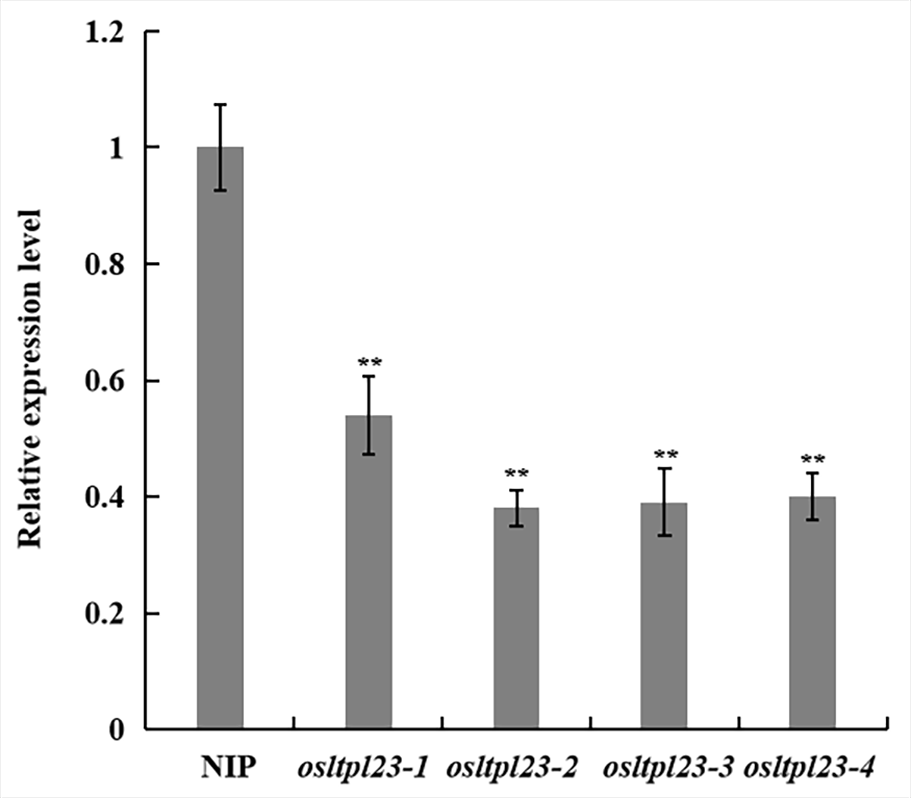

Supplement: Supplementary file 1 [file Image2.TIF]

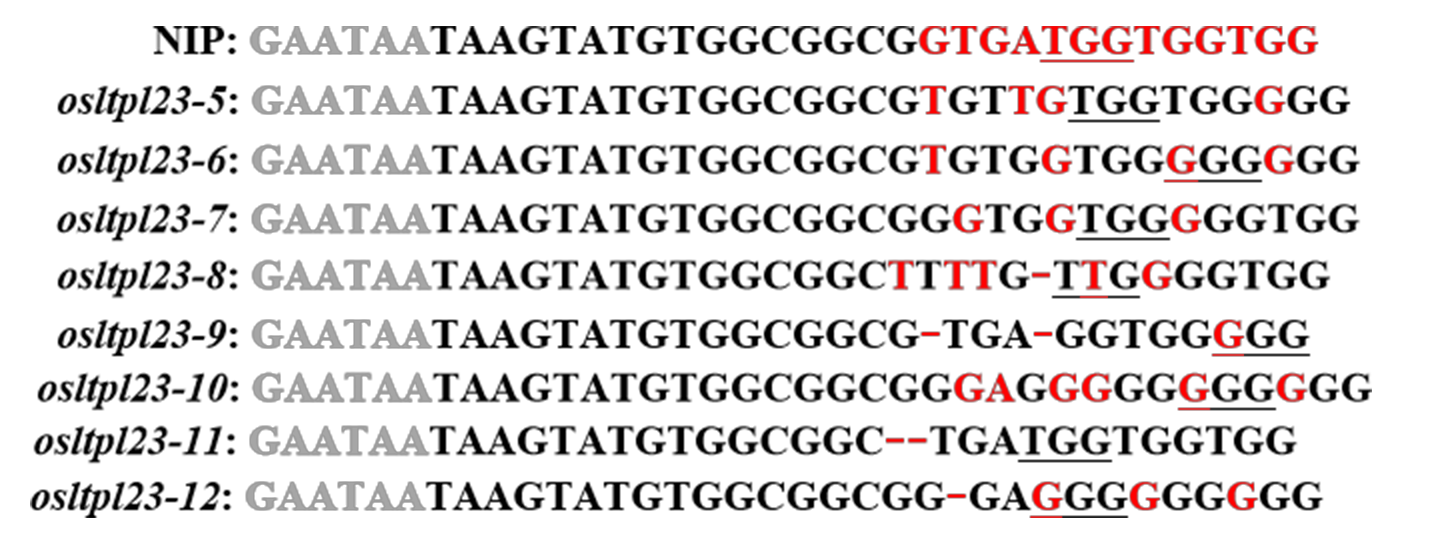

Supplement: Supplementary file 2 [file Image1.TIF]
